# Beaded CoSe_2_-C Nanofibers for High-Performance Lithium–Sulfur Batteries

**DOI:** 10.3390/nano13172492

**Published:** 2023-09-04

**Authors:** Jing Xu, Juan Ao, Yonghui Xie, Yumei Zhou, Xinghui Wang

**Affiliations:** 1 Institute of Micro-Nano Devices and Solar Cells, College of Physics and Information Engineering, Fuzhou University, Fuzhou 350108, China; 15960507831@163.com (J.X.); 15033515321@163.com (J.A.); yonghuix@outlook.com (Y.X.); 201127202@fzu.edu.cn (Y.Z.); 2 Fujian Science & Technology Innovation Laboratory for Optoelectronic Information of China, Fuzhou 350108, China; 3 Jiangsu Collaborative Innovation Center of Photovoltaic Science and Engineering, Changzhou 213000, China

**Keywords:** lithium–sulfur battery, lithium polysulfides, CoSe_2_ nanoparticles, electrospun, carbon nanofiber

## Abstract

Lithium–sulfur (Li-S) batteries are regarded as highly promising energy storage devices due to their high theoretical specific capacity and high energy density. Nevertheless, the commercial application of Li-S batteries is still restricted by poor electrochemical performance. Herein, beaded nanofibers (BNFs) consisting of carbon and CoSe_2_ nanoparticles (CoSe_2_/C BNFs) were prepared by electrospinning combined with carbonization and selenization. Benefitting from the synergistic effect of physical adsorption and chemical catalysis, the CoSe_2_/C BNFs can effectively inhibit the shuttle effect of lithium polysulfides and improve the rate performance and cycle stability of Li-S batteries. The three-dimensional conductive network provides a fast electron and ion transport pathway as well as sufficient space for alleviating the volume change. CoSe_2_ can not only effectively adsorb the lithium polysulfides but also accelerate their conversion reaction. The CoSe_2_/C BNFs-S cathode has a high reversible discharge specific capacity of 919.2 mAh g^−1^ at 0.1 C and presents excellent cycle stability with a low-capacity decay rate of 0.05% per cycle for 600 cycles at 1 C. The combination of the beaded carbon nanofibers and polar metal selenides sheds light on designing high-performance sulfur-based cathodes.

## 1. Introduction

With the rapid development of portable devices and electronic vehicles, it is urgent to develop energy storage devices with higher energy density, lower cost, and better safety [1,2,3,4,5]. Lithium–sulfur (Li-S) batteries have become one of the most promising energy storage systems due to their high theoretical energy density (2600 Wh kg^−1^) [6] coupled with excellent safety features and cost-effectiveness. Sulfur has been intensively investigated as an environmentally friendly cathode material owing to its high theoretical specific capacity (1675 mAh g^−1^) [7], abundant reserves, and economical nature. Despite these advantages, the commercialization of Li-S batteries has been hindered by the following challenges: (1) The poor electrical conductivity of sulfur and Li_2_S/Li_2_S_2_ results in poor rate capabilities of the active material, which makes it difficult for the reduction/oxidation reaction to be fully completed; (2) During the discharge process, the sulfur will react with lithium to form soluble long-chain lithium polysulfides (LiPSs, Li_2_S_n_, 4 ≤ n ≤ 8) at the beginning and then further reduce to insoluble Li_2_S. The soluble LiPSs could dissolve in the electrolyte, named the shuttle effect of LiPSs, resulting in low Coulombic efficiency and fast degradation of the battery capacity along with the cycles; (3) Large volume changes (80%) seriously compromise the stability of the electrode structure during charging and discharging processes, which may cause the collapse of the electrode structure and the loss of the active materials, leading to the deterioration of the cycling stability; (4) The uneven plating/stripping behavior of metal lithium causes the growth of lithium dendrites, which could puncture the separators and cause serious safety accidents [8,9,10,11,12].

There are great efforts to solve the above-mentioned cathode problems through designing various sulfur cathodes with different structures and functions. One of the most common methods is developing carbon-based composite materials to improve the electrochemical performance of the sulfur-based cathodes as nanostructured carbon materials have the advantages of good electrical conductivity, large specific surface areas, and adjustable porous structures. Various nanostructured carbon materials including carbon nanotubes [13], carbon fibers [14], graphene (oxide) [15], and carbon spheres [16] have been employed to effectively improve the overall performance of the Li-S batteries due to the following merits: Firstly, the interconnected conductive skeleton of the carbon-based material can provide a fast channel for electron transmission, guaranteeing a fast reduction/oxidation reaction; Secondly, the large specific surface area of the carbon-based material supplies a higher electrode/electrolyte contact area and a more efficient interaction between the active materials and the Li ions; Thirdly, the adjustable porous structure can accommodate the volume change of the sulfur-based cathode and physically confine the sulfur and LiPSs [17,18]. Therefore, carbon-based materials are ideal host materials to incorporate into Li-S batteries through optimizing electrode conductivity and structure. However, considering the fact that it is difficult to inhibit the shuttle effect of LiPSs since there is a weak interaction between carbon and LiPSs [19,20,21], further enhancing the adsorption capabilities and accelerating the conversion of LiPSs are essential for improving their electrochemical performance.

In recent years, various efforts have been devoted to improving the adsorbtion and electrocatalytic capabilities of carbon-based sulfur cathodes. An emerging strategy is introducing metal or a metal compound into the carbon-based hosts to accelerate the reaction kinetics during charging/discharging and weaken the shuttle effects [22,23,24,25]. He and co-workers discovered that inserting bimetallic CoNi nanoparticles into carbon-based electrodes promotes the chemical immobilization of LiPSs and accelerates their conversion into insoluble Li_2_S [26]. Gao and co-workers designed a bimetallic CoFe alloy decorated interlayer, and the synergistic effect between the Fe and Co species can effectively promote the conversion of LiPSs due to its multiple adsorption and catalytic sites [27]. Wang and co-workers developed a large mesoporous sulfur host, named Co@N-doped carbon nanosheets, which can guarantee fast charge transport, effective physical and chemical trapping, and highly electrocatalytic capabilities for LiPSs, resulting in a much-enhanced electrochemical performance [28]. Liu and co-workers developed a cathode consisting of Co_9_S_8_ nanocrystals and carbon nanotubes encapsulated inside porous N-doped carbon nanofibers. Owing to the multiple polar sites provided by Co_9_S_8_ and N dopant as well as the highly conductive networks provided by the carbon-based composites, the developed cathode demonstrates a high reversible capacity of 1207.7 mAh g^−1^ at 0.1 C and superior cycling performance and rate capability [29]. In general, in comparison with the pure metal, the metal compounds have stronger absorption and electrocatalytic capabilities for LiPSs due to their greater polarity [29].

Among various metal compounds, metal oxides and metal sulfides have been widely used as cathode materials for Li-S batteries, and the corresponding research is relatively well developed [30]. Metal oxides have a strong adsorption effect on LiPSs, but their poor electrical conductivity affects the electron transfer during discharging and charging. Although metal sulfides are superior to metal oxides in terms of electrical conductivity, their adsorption capabilities of LiPSs are poor. Recently, metal selenides, in which selenium is a homologous element of oxygen and sulfur, have attracted extensive attention from research scholars. Metal selenides have similar crystal structures and polar characteristics to metal oxides and metal sulfides but are accompanied by better electrical conductivity. Compared with oxygen and sulfur, selenium atoms have larger atomic radii, higher metallic properties, and lower ionization energies. Therefore, metal selenides can exhibit higher catalytic activity than metal oxides and sulfides [23,31,32]. Xie and co-workers fabricated novel Co_0.85_Se nanoparticles on carbon cloth and embedded them in arrays of nitrogen-doped carbon nanosheets (Co_0.85_Se/NC), which can trap and catalyze lithium polysulfide to accelerate redox kinetics and stabilize the sulfur cathode [23]. Sun and co-workers designed catalytic FeSe_2_ nanoparticles encapsulated with carbon nanoboxes (FeSe_2_@C NBs) to act as multifunctional sulfur hosts, inhibiting polysulfide shuttle effects and accelerating polysulfide redox transformations [31]. Tian and co-workers developed an anion-deficient design of antimony selenide (Sb_2_Se_3−x_) to create a multifunctional LiPSs barrier layer to improve the battery performance [32].

However, the reported metal selenides in the carbon-based hosts have low porosity that cannot provide sufficient space to ease volumetric expansion, resulting in unsatisfied cycling stabilities. Hereby, metal-organic framework (MOF)-derived carbon materials with the advantage of richly tunable porous structures and unique in situ heteroatom doping can confine the LiPSs by both physical domain limitation and chemisorption to enhance their electrochemical performance in Li-S batteries. Nonetheless, the utilization of MOF-derived porous carbon materials doped with heteroatoms still remains insufficient in limiting the shuttle effect of LiPSs. Considering the discussions mentioned above, it is reasonable to use metal selenides as polar adsorbent and electrocatalytic materials in composite with MOF-derived carbon materials to enhance the adsorption of polysulfides, the conductivity of electrodes, and chemical reaction kinetics. Moreover, MOF-derived carbon-based materials are generally discrete nanoparticles, making it challenging to establish effective inter-particle electronic contacts. The utilization of electrospun nanofibers could effectively solve this issue through constructing a one-dimensional electronic interconnection. In addition, the three-dimensional porous features formed by the interconnected nanofibers can alleviate the volume changes of the active materials and enlarge the contact area between the electrolyte and hosts [12,33,34,35]. Overall, although certain progress has been achieved in the exploitation of high-performance sulfur-based cathodes, further effects are still necessary to push the use of Li-S batteries in practical applications [36].

Herein, beaded nanofibers (BNFs) consisting of carbon and CoSe_2_ nanoparticles (CoSe_2_/C BNFs) were synthesized by electrospinning combined with carbonization and selenization. The designed CoSe_2_/C BNF host exhibits the following advantages: (1) CoSe_2_ nanoparticles have strong chemical absorption capability and high catalytic activity for LiPSs, which can effectively inhibit the shuttle effect and accelerate the conversion of LiPSs; (2) The conductive network formed by the carbon nanofibers within the CoSe_2_/C BNFs can reduce the charge transfer impedance of the cathode and buffer the volume change during cycling. As a result, the CoSe_2_/C BNF-S host can present an impressive reversible capacity of 469 mAh g^−1^ after 600 cycles with a low average capacity decay rate of 0.05% per cycle and a low average Coulombic efficiency of 99.1%, much superior to the Co/C BNF and carbon nanofiber (CNF) hosts. These results demonstrate the potential of CoSe_2_/C BNFs as a promising host material for high-performance Li-S batteries.

## 2. Experimental Part

**Synthesis of ZIF67**: The synthesis of ZIF67 was carried out by dissolving 8 mM of cobalt nitrate hexahydrate in 100 mL of methanol in a separate container. In another container, 32 mM of 2-methylimidazole was also dissolved in 100 mL of methanol, which was then slowly added to the cobalt nitrate hexahydrate solution while stirring thoroughly to ensure proper mixing. The resulting mixture was then left to sit at room temperature for 24 h to allow for the formation of ZIF67 crystals. After the 24 h incubation period, the deposits were collected by centrifugation to separate them from the solvent. The collected deposits were then dried at 80 °C in an oven to remove any remaining solvent. The fabricated ZIF67 crystals with purple colour were characterized using various analytical techniques to confirm their purity and composition.

**Synthesis of ZIF67/PAN BNFs**: To synthesize ZIF67/PAN BNFs, an electrospun suspension was prepared by uniformly dispersing 0.75 g ZIF67 and 1.5 g polyacrylonitrile (PAN) in a solvent of 15 mL N, N-dimethylformamide (DMF). The suspension was then loaded into a syringe for electrospinning. The electrospun processes were conducted using a commercial electrospinning machine (TL-Pro, Tong Li Weina Technology Co., Ltd., Shenzhen, China). During electrospinning, the spinning voltage was set to 20 kV and the spinning distance was maintained at 15 cm. Under these conditions, the ZIF67/PAN BNFs were successfully obtained.

**Synthesis of Co/C BNFs**: The ZIF67/PAN BNF composites were annealed inside a tube furnace (BTF-1200C, AnHui BEQ equipment, technology Co., Ltd., Hefei, China) at 230 °C for 2 h and then at 1000 °C for 2 h under Ar gas.

**Synthesis of CoSe_2_/C BNFs**: A piece of Co/C BNFs and an appropriate amount of selenium were placed upstream and in the middle sites of the tube furnace (BTF-1200C, Anhui BEQ equipment technology Co., Ltd., Hefei, China), respectively. The selenization temperature and time were 450 °C and 2 h, respectively.

**Characterization:** The surface morphology of all the samples was characterized with an FEI Helios G4-CX scanning electron microscope. X-ray diffraction (XRD) measurements were carried out using a Rigaku SmartLab with Cu Kα as the X-ray source. The electrochemical performance of the cathode was evaluated using the coin cells (CR2032) that assembled in a Mikrouna glove box filled with Ar (O_2_ < 0.1 ppm, H_2_O < 0.1 ppm). The sulfur active materials were realized by dropping 10 μL of 0.6 M Li_2_S_6_ catholyte on self-supported CNF, Co/C BNFs, and CoSe_2_/C BNFs with a size of 1 × 1 cm^2^, and the corresponding sulfur loading was around 1.15 mg cm^−2^. The galvanostatic charging/discharge, long cycling performance, and rate performance were measured with a Neware battery testing system (BST-4000). Cyclic voltammetry (CV) and electrochemical impedance spectroscopy (EIS) were tested using a Biologic electrochemical workstation (SP-200).

## 3. Results and Discussion

**Figure 1:**
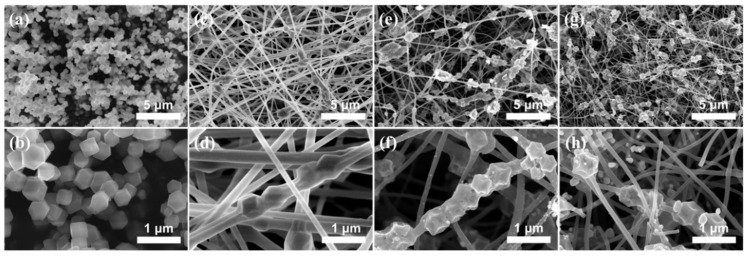
The low- and high-magnification SEM images of (**a**,**b**) ZIF67; (**c**,**d**) ZIF67/PAN BNFs; (**e**,**f**) Co/C BNFs; and (**g**,**h**) CoSe_2_/C BNFs.

The morphological changes in the samples during the fabrication processes are shown in Figure 1. ZIF67 was first prepared by solution method, which exhibits a uniform dodecahedral structure with a size of about 300 nm (Figure 1a,b). Then, ZIF67/PAN BNFs with smooth surfaces (Figure 1c,d) were obtained via electrospinning a suspension containing ZIF67 and PAN. As shown in Figure 1d, some ZIF67 dodecahedral particles can be found inside the nanofibers, suggesting the successful electrospinning of the composites. After the heat treatment, the ZIF67 and PAN are converted into Co/C composites. The resulting Co/C BNFs exhibit an interactive network structure constructed from rough and porous fibers (Figure 1e,f) that provides the effective contact surfaces between the electrode and the active material to enhance the electrochemical reaction. Subsequently, the fiber-like structure remains the same after selenization, the CoSe_2_/C BNF fibers decorate with uniformly-dispersed CoSe_2_ nanoparticles (Figure 1g,h) on the surface, which originate from the reaction of the Co and Se metal. As reported, the CoSe_2_ nanoparticles could serve as an effective trapping agent and a catalyst for LiPSs [12,37].

**Figure 2:**
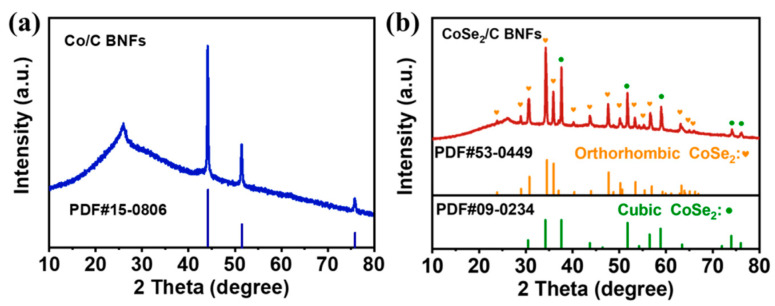
XRD images of (**a**) Co/C BNFs and (**b**) CoSe_2_/C BNFs.

For the purpose of revealing the phase composition, XRD characterizations were performed on the Co/C BNFs and CoSe_2_/C BNFs. As shown in Figure 2a, Co/C BNFs exhibit three sharp peaks located at 44.1°, 51.4° and 75.8°, which can be assigned to the (111), (200), and (220) crystal faces of the cubic metal Co (PDF#15-0806), respectively. In addition, the wide diffraction peak located at 26° is consistent with that of carbon materials [38,39,40]. After selenization, the main characteristic peaks of the CoSe_2_/C BNFs in the XRD pattern (Figure 2b) can be assigned to the mixture of cubic CoSe_2_ (PDF#09-0234) and orthorhombic CoSe_2_ (PDF#53-0449). Specially, the main peaks located at 37.6° and 51.7° correspond to the (211) and (311) crystal faces of cubic CoSe_2_, while the peaks at 34.5° and 35.9° can be indexed as the (111) and (120) crystal faces of orthorhombic CoSe_2_. There are no other characteristic peaks of Co that can be found from the XRD patterns after selenization, suggesting that the conversion of Co into CoSe_2_ is complete.

**Figure 3:**
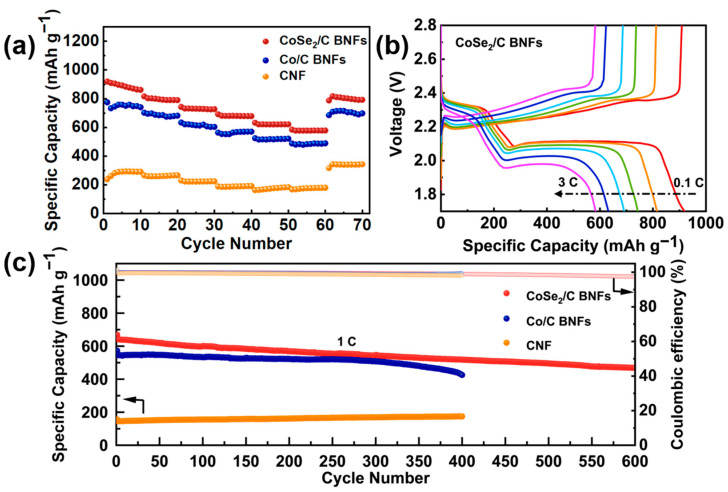
(**a**) Rate performance of CNF-S, Co/C BNF-S, and CoSe_2_/C BNF-S electrodes from 0.1 to 3 C; (**b**) The representative charge and discharge curves of the CoSe_2_/C BNF-S electrode at various current rates; (**c**) The long-term cycling performance of CNF-S, Co/C BNF-S, and CoSe_2_/C BNF-S electrodes at a current rate of 1 C.

In order to evaluate the electrochemical performance, CNF-S, Co/C BNF-S, and CoSe_2_/C BNF-S electrodes were assembled into coin-type Li-S batteries with Li_2_S_6_ catholyte to provide the sulfur active materials. Figure 3a compares the rate performance of the CNF-S, Co/C BNF-S, and CoSe_2_/C BNF-S electrodes at current rates ranging from 0.1 to 3 C. Obviously, the capacities of the CNF-S and Co/C BNF-S electrodes are remarkably smaller than that of the CoSe_2_/C BNF-S electrode at all rates, which indicates that the designed metal selenide composite material has greater advantages than metal composite material. The discharge specific capacities of the CoSe_2_/C BNF-S electrodes are 919.2, 815.6, 742.8, 691.2, 631.8, and 583.8 mAh g^−1^ at current rates of 0.1, 0.2, 0.5, 1, 2, and 3 C, respectively. In contrast, the CNF-S electrode delivers discharge specific capacities of 238.8, 265.8, 230.0, 188.8, 162.9, and 168.0 mAh g^−1^ respectively, while the Co/C BNF-S electrode preserves discharge specific capacities of 774.3, 701.7, 634.0, 563.2, 524.7, and 487.2 mAh g^−1^, respectively. More importantly, the specific capacity of the CoSe_2_/C BNF-S electrode is restored to 787.1 mAh g^−1^ when the current rate is returned to 0.1 C, demonstrating a good resistance of the electrode to current rate changes. These results indicate that CoSe_2_/C BNFs-S exhibit fast sulfur redox kinetics and highly reversible electrochemical reaction capabilities. Figure 3b presents the representative charge and discharge curves of the CoSe_2_/C BNF-S electrode at various rates. One can see that they have the typical charge and discharge plateaus of the Li-S battery, and its voltage platform remains stable even under a high current rate of 3 C. With the rise in current rate, the charge and discharge plateaus exhibit a tendency to increase and decrease, respectively, indicating an escalated level of polarization. The augmentation of polarization with increasing current rate primarily stemmed from ohmic resistance and heightened activation polarization.

With the intention of evaluating the cyclic stability and Coulombic efficiency of CNF-S, Co/C BNF-S and CoSe_2_/C BNF-S electrodes, the long-term cycling performance of each electrode was studied at a current rate of 1 C. As shown in Figure 3c, the CoSe_2_/C BNF-S electrode achieves a high discharge specific capacity of 668.6 mAh g^−1^ in the initial cycle, which is much higher than that of the Co/C BNF-S (571.9 mAh g^−1^) and CNF-S (156.5 mAh g^−1^) electrodes. The discharge specific capacity of the CoSe_2_/C BNF-S electrode remains at 469 mAh g^−1^ after 600 cycles, with an average capacity decay rate of 0.05% per cycle and an average Coulombic efficiency of 99.1%, indicating that the CoSe_2_/C BNF-S electrode exhibits stable long-term cycling stabilities. In comparison with CNF-S and Co/C BNF-S, the CoSe_2_/C BNF-S electrode has superior rate performance and cycling performance, which can be attributed to CoSe_2_ nanoparticles that can absorb and accelerate the redox reaction of LiPSs [41,42].

**Figure 4:**
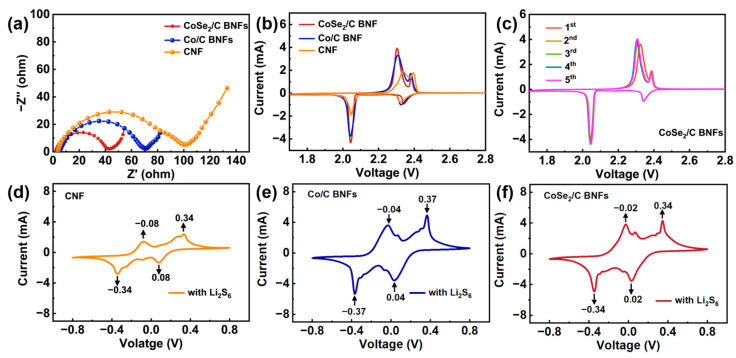
(**a**) EIS profiles and (**b**) CV curves of CNFs-S, Co/C BNFs-S, CoSe_2_/C BNFs-S; (**c**) Initial five CV profiles of CoSe_2_/C BNFs-S at 0.1 mV s^−1^; CV curves of (**d**) CNF-S, (**e**) Co/C BNF-S, and (**f**) CoSe_2_/C BNF-S symmetric batteries at 0.5 mV s^−1^.

In order to further explore the intrinsic mechanisms behind the enhanced electrochemical performance of the CoSe_2_/C BNF-S electrode, EIS was utilized to gain insight into the charge transfer properties of CNFs-S, Co/C BNFs-S, and CoSe_2_/C BNFs-S. Figure 4a shows the Nyquist plots of the different electrodes at the initial state of the half-cell, all of which are presented with a semicircle in the higher frequency range and a diagonal line in the lower frequency region. The diameter of the semicircle in the higher frequency range corresponds to the charge transfer resistance, while the diagonal line in the lower frequency region signifies the semi-infinite Warburg diffusion of Li^+^ within the electrode [37]. From the EIS results, it can be inferred that the charge transfer resistance of the CoSe_2_/C BNF-S electrode is significantly smaller than that of the Co/C BNF-S electrode and CNF-S electrode, suggesting that the CoSe_2_ could accelerate the reaction kinetics of the LiPSs conversion.

To reveal the reaction mechanism of the electrodes, the CV profiles of the CNF-S, Co/C BNF-S and CoSe_2_/C BNF-S batteries were conducted in the voltage window from 1.7 to 2.8 V at a scan rate of 0.1 mV s^−1^. As shown in Figure 4b, all the electrodes exhibit cathodic peaks at around 2.34 and 2.04 V which correspond to the reduction of sulfur to long-chain LiPSs (Li_2_S_n_, 4 ≤ n ≤ 8) and further to Li_2_S_2_/Li_2_S, respectively. There are two anodic peaks at around 2.3 and 2.4 V that can be assigned to the oxidation of Li_2_S/Li_2_S_2_ to long-chain LiPSs and further to sulfur. These results are in general agreement with the reported CV profiles [23,37,43,44]. Moreover, the CoSe_2_/C BNF-S battery has a larger area under the CV curve than that of the CNF-S and Co/C BNF-S batteries, indicating a higher specific capacity, which is consistent with the rate and cycle performance. Furthermore, the initial five CV profiles of the CoSe_2_/C BNFs-S are shown in Figure 4c. It is noteworthy that the CV curves overlap very well after the initial cycle, which again indicates the good electrochemical reversibility of the CoSe_2_/C BNF-S electrode.

To further verify the catalytic effect of CoSe_2_/C BNFs, the CV profiles of CNF-S, Co/C BNF-S, and CoSe_2_/C BNF-S symmetrical cells were investigated in the presence of Li_2_S_6_ electrolytes at a scan rate of 0.5 mV s^−1^. As shown in Figure 4d–f, the CoSe_2_/C BNF-S cell demonstrates superior catalytic activity for LiPS conversion compared to the CNF-S and Co/C BNF-S cells, as evidenced by its smallest voltage difference between the redox peaks. Therefore, CoSe_2_/C BNFs could accelerate the reaction kinetics of the LiPS conversion, making them a promising candidate for developing high-performance Li-S batteries.

The results of this study suggest that CoSe_2_/C BNFs have the ability to significantly reduce the shuttle effect of LiPSs, leading to improved long cycle and rate performances of Li-S batteries. These enhanced electrochemical properties of CoSe_2_/C BNFs can be attributed to the following aspects: Firstly, the three-dimensional conductive network of CoSe_2_/C BNFs facilitates rapid electron transport, thereby reducing resistance in the battery system and improving the rate performance; Secondly, the numerous pores formed by the conductive network are capable of increasing the contact area of the electrolyte/active materials as well as accommodating volume changes during cycling, which can facilitate fast ion transport and improve the overall stability and durability of the system, respectively; Thirdly, CoSe_2_ is known for its ability to capture and catalyze the conversion of LiPSs. This capability enables CoSe_2_/C BNFs to effectively inhibit the shuttle effect of LiPSs, leading to higher sulfur utilization rates. As a result, CoSe_2_/C BNF-based Li-S batteries exhibit excellent rate capabilities and cycling performance. Overall, the study highlights the potential benefits of CoSe_2_/C BNFs as a promising catalyst material for developing high-performance Li-S batteries. By enhancing the electrochemical properties of the system, CoSe_2_/C BNFs can help overcome some of the challenges associated with the practical use of Li-S batteries, making them a viable alternative to other energy storage technologies.

## 4. Conclusions

In summary, we developed three-dimensional, free-standing, and multifunctional porous beaded nanofibers consisting of carbon and CoSe_2_ nanoparticles that can be applied as high-performance sulfur hosts for Li-S batteries. The three-dimensional conductive network not only plays a crucial role in limiting the shuttle effect of polysulfides by physically limiting the domain, but also provides a fast channel for Li^+^ transport. In particular, the composite bifunctional CoSe_2_ nanoparticles not only strengthen the chemisorption of polysulfides, but also accelerate the chemical reaction kinetics of the LiPS conversion. Based on the physical and chemical multi-trapping effect of the CoSe_2_/C BNF composite on polysulfides, the CoSe_2_/C BNF-S anode exhibits excellent rate and cycle performance. The CoSe_2_/C BNF-S electrode could deliver a high reversible discharge specific capacity (919.2 mAh g^−1^ at 0.1 C), excellent rate performance (583.8 mAh g^−1^ at 3 C), and stable long-term cycling stability (a discharge specific capacity of 469 mAh g^−1^ after 600 cycles at 1 C, corresponding to a decay rate of about 0.05% per cycle), which are superior compared to the control sample of pure CNFs and Co/C BNFs-S. The design of beaded nanofibers consisting of carbon and metal selenides has inspired materials engineering for high-performance sulfur cathodes. Therefore, this work not only improves the electrochemical performance of Li-S batteries, but also provides a new idea for optimizing the design of novel electrodes for various energy storage devices.

## Data Availability

Raw data for the article are available, pending request to the corresponding authors.
